# Towards a Thermodynamical Deep-Learning-Vision-Based Flexible Robotic Cell for Circular Healthcare

**DOI:** 10.1007/s43615-025-00532-4

**Published:** 2025-04-02

**Authors:** Federico Zocco, Denis Sleath, Shahin Rahimifard

**Affiliations:** Centre for Sustainable Manufacturing and Recycling Technologies, Wolfson School of Mechanical, Electrical and Manufacturing Engineering, Loughborough University, Ashby Road, Loughborough, LE11 3TU England UK

**Keywords:** Robotic waste sorting, Robotic disassembly, Medical devices, Circular robotics, Circular intelligence

## Abstract

The dependence on finite reserves of raw materials and the generation of waste are two unsolved problems of the traditional linear economy. Healthcare, as a major sector of any nation, is currently facing them. In addition, the reprocessing of healthcare waste poses humans at risk of contamination. Another open issue is that circular economy, which is a paradigm that is being proposed to address material supply uncertainties and waste generation, still lacks physics-based modeling approaches that enable the design and analysis of circular flows of materials. Hence, in this paper, first we report on the on-going development of a flexible robotic cell enabled by deep-learning vision for automating three main tasks in a circular healthcare, namely, resources mapping and quantification, disassembly, and waste sorting of small medical devices. Second, we combine compartmental dynamical thermodynamics with the mechanics of robots to integrate robotics into a system-level perspective. Our thermodynamic framework is a step forward in defining the theoretical foundations of circular material flow designs because it enhances material flow analysis (MFA) by adding dynamical energy balances to the usual mass balances and by leveraging dynamical systems theory. Third, we propose two circularity indicators by leveraging our thermodynamic framework and graph theory. While our initial set-up of the robotic cell is for reprocessing glucose meters and inhalers, other medical devices can be considered after making the proper adaptations; in addition, it can switch from sorting to disassembly to resources mapping and quantification, or run them in parallel. Our thermodynamic systemic modeling framework involves more physics and system dynamics than MFA, and hence, can yield the needed improvements in model accuracy and reproducibility at the cost of extra complexity. Finally, the proposed circularity indicators can help healthcare chain managers in assessing whether the robotic cell can process the input stream of materials within the desired time and with the desired level of separation at the output material flow. Software and a demo video are publicly available.

## Introduction

The OECD global material resources outlook to 2060 states that the global primary materials use is projected to almost double from 89 gigatonnes in 2017 to 167 gigatonnes in 2060 [1]. The strongest growth in materials use is expected to occur in emerging and developing countries; even in the OECD countries the growth should be between 1% to 2% per year on average [1]. Since most of the materials are finite resources, the transition to a more circular and resource efficient economy is essential to prevent future material supply shortages [2].

The second benefit of implementing a more circular economy, i.e., an economy based on reducing, reusing, repairing, and recycling, is that waste and pollution rates will be reduced since products and materials are kept in use for longer while output flows from processes and households are recirculated within the economy [3]. For example, in healthcare, the UK NHS alone produces 156,000 tonnes of clinical waste each year [4], whose recovery must be both economically viable and free from contamination risks. According to [5], the implementation of effective measures to eliminate waste represents an opportunity to reduce US healthcare expenditure, specifically it was estimated a potential reduction of 25% in the total cost of waste. In 2017, China’s ban on its import of most plastic waste has also motivated developed countries to implement reprocessing approaches locally [6].

In addition to mitigating waste generation, it has been argued that circular practices could help the UK NHS to meet its target to be net zero by 2040 [7]. Indeed, circularity can reduce the carbon emissions of the healthcare sector by keeping products in use for longer, and hence, reduce mining activities and production of raw materials [7]. The exceptions are single-use medical devices used for very high contamination-risk treatments, which are unlikely to be replaced by reusable devices due to unacceptable levels of risk to the safety of patients [7]. An example of these single-use devices are those employed during the COVID-19 pandemic [8]. Extending the life-cycle of products and materials will also reduce NHS carbon emissions resulting from the incineration of medical waste [7].

In October 2024, the UK government published the “Design for Life” roadmap, an initiative with the goal of delivering a circular approach to medical technologies by designing, procuring, and processing medical products in order to be reused, remanufactured, and recycled [9]. Among the major problems identified in [9] are the fact that linear products are currently the default choice, stakeholders are insufficiently incentivised to adopt circular practices, the current physical and digital infrastructures restrict the progress of circular solutions, and the innovation ecosystem does not aim to increase circularity. Examples of the actions and outputs addressing these problems will be to quantify the volume capacity requirements to be satisfied by a circular healthcare sector, identify digital solutions to speed-up the implementation of circularity, and develop a decontamination infrastructure along with effective materials recovery and recycling strategies [9].

To enhance a domestic implementation of circular material flows in healthcare, in this paper we make the following main contributions:

- Since autonomous reprocessing systems can reduce both costs and contamination risks, we report on the on-going development of a *flexible robotic cell for reprocessing small medical devices* such as glucose meters and inhalers. Minor layout changes can set the cell for waste sorting, disassembly, resources mapping and quantification, or run them in parallel (see Sections 2.2 and 3.1).
- So far, the theory of robot design and control is not integrated into the design of whole supply and recovery chains. To address this gap and improve the supply-recovery chain models in accuracy and reproducibility, we *integrate the standard form of robot dynamics into a systemic framework* by leveraging compartmental dynamical thermodynamics [10, 11] (see Section 2.1).
- We provide two *circularity indicators of the robotic cell* to help decision making about supply-recovery chains in healthcare (see Sections 2.3 and 3.2).
- From a high-level perspective, this paper coherently intersects compartmental dynamical thermodynamics, robotics theory, deep-learning computer vision, and graph theory to *advance both the theoretical foundations and the practical implementation of a circular economy* for small medical devices.

Related work is as follows. Robotic disassembly to recover rare Earth materials from electronic components of electric vehicles was proposed by [12], while disassembly sequence planning was addressed by [13, 14]. An extensive book on disassembly is [15]. Kiyokawa et al. [16] recently reviewed the most advanced robot functionalities discussing how to extend the flexibility of traditional waste sorting facilities, which target only a limited range of items. As it will be shown, we also seek flexibility in our robotic cell. Waste sorting of clinical waste was proposed in 2020 by [17], whereas [18] and [19] focused on textiles and urban waste, respectively.

An important role in robotic disassembly and waste sorting is played by the gripper used to handle the objects. It is widely recognized in robotics that rigid structures are less suitable than *soft* ones to work in unstructured environments. As a consequence, the trend in robotic manipulation are soft grippers [20], i.e., grippers whose components are made of deformable materials. While the manipulation task in waste sorting can be significantly simplified using a pick-and-toss approach [19], dexterous soft robotic hands are needed in disassembly operations to handle objects of various, and unpredictable, shapes. For example, Achilli *et al.* [21] proposed a soft gripper for the waste industry whose design has no electronic parts and the number of parts made of non-recyclable materials is minimized. Along with softness, another important property of adaptive grasping mechanisms is *under-actuation*, that is, having fewer actuators than degrees of freedom. Indeed, under-actuated mechanisms have shown promising performance in self-adapting grasping tasks [22–24].

One of the latest improvements in robot functionalities is brought by the recent breakthroughs in machine learning and consists of more accurate and generalizing vision models for visual servoing, i.e., for controlling the robot motion via real-time processing of images acquired by cameras [25–28]. Effective visual servoing could leverage the systems proposed by [29–31] for general and flexible material, object, and part recognition. Generally speaking, flexible robotic operations require the synergy of the soft grippers discussed above with vision algorithms able to recognize items with different colors, shapes, textures, and components, in real-time and with uncertain conditions of dirt and light. The key property of such vision algorithms is *generalization*, i.e., the ability to infer useful properties of sample images that have never been processed by the algorithm before [32]. Generalization is the opposite of *overfitting*, which is the behavior of a vision system that is exceptionally accurate in inferring information from training samples, but its performance decreases drastically when processing unseen images [33]. Making vision systems able to generalize is central in computer vision and one of the main strategies to enhance generalization is to train the model on a large amount of data. To aid in this endeavor, large datasets with images of waste items in different environments have been collected, annotated, and made publicly available [34–36].

One of the most promising directions to achieve flexible vision-based robotic manipulation is to perform the learning phase with a reinforcement-learning algorithm that continuously collects images from the scene and on-line learns to achieve the desired goal of the system as done in [37]. To date, reinforcement-learning algorithms have been shown to work for many simulated problems, but achieving a similar performance on real-world tasks remains an open area of research due to fundamental assumptions in the algorithms that are often not valid in practice [38].

To date, MFA is a key methodology to assess material flow circularity; essentially, it is based on the principle of mass conservation and on the analysis of real material stock and flow data [39]. As it was shown in [10, 40] and further in this paper, our thermodynamic framework enhances MFA in two aspects: first, it adds dynamical energy balances [11] to the usual mass balances; and second, it is underpinned by continuous- and discrete-time dynamical systems rather than by data-analysis, and hence, it is more accurate in simulating what-if scenarios, in which real data are missing (see [41] and [42] for an introduction and an advanced book on dynamical systems, respectively). The lack of real data is very common in circular economy research since circular material flows are still rare in reality.

Throughout the paper, vectors and matrices are indicated with bold lower-case and upper-case letters, respectively, while sets are indicated with upper-case calligraphic letters.

The paper is structured as follows: Section “Methods” details the robotic cell design, the thermodynamic framework, and the circularity indicators; then, Section “Results and Discussion” shows and discusses the results; finally, Section “Conclusions” concludes.

## Methods

This section begins by integrating the theory of robot mechanics into the systemic modeling framework of thermodynamical material networks (TMNs) [10], which is required to address system-level questions such as those arising in circular economy. Then, it describes the robotic cell we are developing and proposes two circularity indicators to assess its performance.

### Integration of Robotics into a Systemic Framework

#### Principles of TMNs and Circularity

The definition of a TMN results from the combination of graph theory [43] and compartmental dynamical thermodynamics [11]: the former is a well-established approach for analyzing networks (e.g., hydraulic networks [44], electrical networks [45], and epidemic networks [46]), while the latter is a formalism that generalizes the mature design technique of water and thermal systems [47]. Specifically, consider the Rankine cycle invented by the Scottish engineer William J. M. Rankine in 1859. It is an idealized thermodynamic cycle covered in introductory courses in thermal engineering and used for simplified quantitative analysis of real thermal plants (see, for example, Section 8.2 in [48]). The Rankine cycle is made of four main components, i.e., thermodynamic compartments, connected in series: a turbine, a condenser, a pump, and a boiler; these four compartments are connected through four pipelines that transport the working fluid from a compartment to the next in a closed loop. Since the Rankine cycle is a closed loop, we can say that it is a *circular* system and that the working fluid has a circular flow. As detailed in [48], the modeling approach of the Rankine cycle consists of applying the mass conservation principle along with the first and second laws of thermodynamics to each compartment one at a time. The idea underpinning TMNs is to leverage the generality of thermodynamics [49] by applying the design approach of the Rankine cycle to any material flow, whether it is a solid or a fluid. This results in approaching the design of supply-recovery chains by applying the mass conservation principle and the laws of thermodynamics to each stage of the life-cycle one at a time [10].

To note that, in this paper, we go straight to the definition of the discrete-time mass-flow matrix, which assumes familiarity with the preliminary definitions covered by [10, 40].

##### Definition 1

(Discrete-time mass-flow matrix). Given a TMN

1 $$\begin{aligned} \begin{gathered} \mathcal {N} = \left\{ c^1_{1,1}, \dots, c^{k_v}_{k_v,k_v}, \dots, c^{n_v}_{n_v,n_v}, \right. \\ \left. c^{n_v+1}_{i_{n_v+1},j_{n_v+1}}, \dots, c^{n_v+k_a}_{i_{n_v+k_a},j_{n_v+k_a}}, \dots, c^{n_c}_{i_{n_c},j_{n_c}}\right\}, \end{gathered} \end{aligned}$$

the *discrete-time mass-flow matrix* $\varvec{\Gamma }(\mathcal {N}; n)$ associated with the network Eq. 1 is

2 $$\begin{aligned} \begin{aligned} \varvec{\Gamma }(\mathcal {N}; n)&= \begin{bmatrix}{} \gamma _{1,1}(n+1) & \dots & \gamma _{1,n_v}(n+1) \\ \vdots & \ddots & \vdots \\ \gamma _{n_v,1}(n+1) & \dots & \gamma _{n_v,n_v}(n+1) \end{bmatrix} \\&= \begin{bmatrix}{}m_1(n+1) & m_{1,2}(n+1) & \dots & m_{1,n_v}(n+1) \\ m_{2,1}(n+1) & m_2(n+1) & \dots & m_{2,n_v}(n+1) \\ \vdots & \vdots & \ddots & \vdots \\ m_{n_v,1}(n+1) & m_{n_v,2}(n+1) & \dots & m_{n_v}(n+1) \end{bmatrix} \end{aligned} \end{aligned}$$

where $n \in \overline{\mathbb {Z}}_+$, $\varvec{\Gamma }(\mathcal {N}; n) \in \mathbb {R}^{n_v \, \times \, n_v}$, $\overline{\mathbb {Z}}_+$ is the set of non-negative integers, $n_v$ is the number of vertices, $n_c$ is the total number of compartments, the entries along the diagonal are the weights of the vertex-compartments $c^{k_v}_{k_v,k_v} \in \mathcal {R}$ (i.e., mass stocks), with $\mathcal {R} \subseteq \mathcal {N}$ the subset of compartments that *store*, *transform*, or *use* the target material, and whose off-diagonal entries are the weights of the arc-compartments $c^k_{i,j} \in \mathcal {T}$ (i.e., masses that move between the vertices), with $\mathcal {T} \subseteq \mathcal {N}$ the subset of compartments that *move* the target material between the compartments belonging to $\mathcal {R} \subseteq \mathcal {N}$.

Conceiving the material life-cycle as a sequence of thermodynamic compartments that transport and transform the material leads to the following definition of circularity.

##### Definition 2

([10]). The flow of a material $\beta$ is *thermodynamically* circular if there exists an ordered sequence of thermodynamic compartments

3 $$\begin{aligned} \phi = \left( c_{1,1}^1, \dots, c_{i,j}^k, \dots, c_{1,1}^1\right) \end{aligned}$$

processing $\beta$ which begins and ends in $c_{1,1}^1$. More generally, the flow of the material set $\mathcal {B} = \{\beta _1, \dots, \beta _k, \dots, \beta _{n_{\beta }}\}$ is thermodynamically circular if there exists an ordered sequence $\phi$ processing $\mathcal {B}$.

#### Robot as a Thermodynamic Compartment

The previous section has defined a systemic modeling framework in which the material flow results from a network of thermodynamic compartments connected by the exchange of materials. Therefore, if we demonstrate that a robot can be seen as a thermodynamic compartment of such a network, we achieve the integration of robotics theory into this systemic framework so that we can design industrial robotic operations as part of supply and recovery chains (i.e., networks) instead of stand alone. A systemic framework is essential to address circular economy questions as they are systemic in their nature. Such an integration of robotics theory is achieved with the following proposition.

##### Proposition 1

The standard form of robot dynamics (see Ch. 7 in [50])

4 $$\begin{aligned} \begin{gathered} \sum _{j=1}^\alpha b_{ij}(\varvec{q})\ddot{q}_j + \sum _{j=1}^\alpha \sum _{k=1}^\alpha h_{ijk}(\varvec{q})\dot{q}_k\dot{q}_j \\ + g_i(\varvec{q}) = \xi _i, \quad i = 1, \dots, \alpha, \end{gathered} \end{aligned}$$

can be derived from the dynamical form of the first principle of thermodynamics

5 $$\begin{aligned} \frac{\text {d}E}{\text {d}{t}} = \dot{Q} - \dot{W}, \end{aligned}$$

where *E* is the total energy of the robotic manipulator, $\dot{Q}$ is the heat flow exchanged between the manipulator and the surroundings, $\dot{W}$ is the work flow exchanged between the manipulator and the surroundings, $\alpha$ is the number of rigid links of the robotic manipulator, $\varvec{q}$ is the vector of generalized coordinates, $q_j$ is the *j*-th element of $\varvec{q}$, $\varvec{\xi }$ is the vector of generalized forces, and where $b_{ij}(\varvec{q})$, $h_{ijk}(\varvec{q})$, and $g_i(\varvec{q})$ are coefficients that take into account the gravity, the Coriolis effect, the centrifugal effect, and the inertias of the links.

It follows from Proposition 1 that a robot can be seen as a thermodynamic compartment, and hence, its modeling and control can be embedded into the design of a TMN [10]. As explained above when discussing the Rankine cycle, the TMN methodology conceives supply-recovery chains as a sequence of thermodynamic compartments; Proposition 1 demonstrates that a robot is a thermodynamic compartment, and hence, it can be integrated into a TMN (refer to Fig. 1 in [51] for a graphical representation of the concept). Thanks to the generality of thermodynamics [49], the TMN methodology is general. The healthcare sector is the area of application considered in this work.

##### Proof

Consider the dynamical form of the first principle of thermodynamics Eq. 5 with the total energy contributions developed as $E = K + U + P$, where *U* is the internal energy, *K* is the kinetic energy, and *P* is the potential energy of the system under study. Since *U* and the heat flow $\dot{Q}$ are neglected in solid mechanics, Eq. 5 reduces to

6 $$\begin{aligned} \frac{\text {d}}{\text {d}{t}}(K + P) = - \dot{W}. \end{aligned}$$

Taking the partial derivative on both sides of Eq. 6 with respect to $\varvec{\dot{q}}$ and transposing the resulting equation yields

7 $$\begin{aligned} \frac{\text {d}}{\text {d}{t}}\left[ \frac{\partial (K + P)}{\partial \dot{\varvec{q}}}\right] ^\top = - \left( \frac{\partial \dot{W}}{\partial \dot{\varvec{q}}}\right) ^\top. \end{aligned}$$

Recalling the Lagrangian function

8 $$\begin{aligned} L = K - P \end{aligned}$$

and the fact that $\partial P / \partial \dot{\varvec{q}} = 0$, Eq. 7 can be written as

9 $$\begin{aligned} \frac{\text {d}}{\text {d}{t}}\left( \frac{\partial L}{\partial \dot{\varvec{q}}}\right) ^\top = - \left( \frac{\partial \dot{W}}{\partial \dot{\varvec{q}}}\right) ^\top. \end{aligned}$$

Note now that the left-hand side of the formulation of the Lagrange’s equations of motion

10 $$\begin{aligned} \frac{\text {d}}{\text {d}{t}}\left( \frac{\partial L}{\partial \dot{\varvec{q}}}\right) ^\top = \varvec{\xi } + \left( \frac{\partial L}{\partial \varvec{q}} \right) ^\top \end{aligned}$$

is equal to the left-hand side of Eq. 9. Hence, the right-hand sides of Eqs. 10 and 9 are also the same. Thus, Eq. 9 can be written as Eq. 10, and hence, the Lagrange’s equations of motion Eq. 10 can be seen as a derivation of the power balance Eq. 5. To complete the proof, we will now show that Eq. 10 can be written as the standard form of robot dynamics Eq. 4.

Consider a manipulator with $\alpha$ rigid links. The total kinetic energy is the sum of the kinetic energy of all the links and all the motors (see Ch. 7 in [50]), that is,

11 $$\begin{aligned} K = \sum _{i=1}^\alpha (K_{\text {l}_i} + K_{\text {m}_i}), \end{aligned}$$

where $K_{\text {l}_i}$ is the kinetic energy of the *i*-th link and $K_{\text {m}_i}$ is the kinetic energy of the motor actuating Joint *i*. The kinetic energy contribution of Link *i* is

12 $$\begin{aligned} K_{\text {l}_i} = \frac{1}{2} \int _{V_{\text {l}_i}} \dot{\varvec{p}}^{*\top }_i \dot{\varvec{p}}^*_i \rho \text {d}V, \end{aligned}$$

where $\dot{\varvec{p}}^*_i$ denotes the linear velocity vector and $\rho$ is the density of the elementary particle of volume $\text {d}V$; $V_{\text {l}_i}$ is the volume of Link *i*. Be $\varvec{p}_i^*$ the position vector of the elementary particle and be $\varvec{p}_{\text {l}_i}$ the position vector of the link centre of mass expressed in the base frame. Hence, we can write

13 $$\begin{aligned} \varvec{r}_i = \varvec{p}^*_i - \varvec{p}_{\text {l}_i} \end{aligned}$$

and, thus, the link particle velocity can be written as

14 $$\begin{aligned} \dot{\varvec{p}}^*_i = \dot{\varvec{p}}_{\text {l}_i} + \varvec{\omega }_i \times \varvec{r}_i = \dot{\varvec{p}}_{\text {l}_i} + \varvec{S}(\varvec{\omega }_i)\varvec{r}_i, \end{aligned}$$

with $\dot{\varvec{p}}_{\text {l}_i}$ the linear velocity of the center of mass, $\varvec{\omega }_i$ the angular velocity of the *i*-th link, and $\varvec{S}(\cdot )$ a matrix operator (see Ch. 7 in [50]). By substituting Eqs. 14 into 12, we have that

15 $$\begin{aligned} K_{\text {l}_i} = \frac{1}{2} \int _{V_{\text {l}_i}} \left( \dot{\varvec{p}}_{\text {l}_i} + \varvec{S}(\varvec{\omega }_i)\varvec{r}_i\right) ^\top \left( \dot{\varvec{p}}_{\text {l}_i} + \varvec{S}(\varvec{\omega }_i)\varvec{r}_i\right) \rho \text {d}V, \end{aligned}$$

which contains three contributions to the velocity: the translational term, the rotational term, and the mutual term. By introducing the Jacobian columns relative to the joint velocities up to the Link *i* with the matrices $J^{(\text {l}_i)}_{\text {P}}$ and $J^{(\text {l}_i)}_{\text {O}}$, the kinetic energy of Link *i* in Eq. 15 can be written as a function of $\varvec{\varvec{q}}$ as

16 $$\begin{aligned} K_{\text {l}_i} = \frac{1}{2} m_{\text {l}_i} \dot{\varvec{q}}^\top J^{(\text {l}_i)\top }_{\text {P}} J^{(\text {l}_i)}_{\text {P}} \dot{\varvec{q}} + \frac{1}{2} \dot{\varvec{q}}^\top J^{(\text {l}_i)\top }_{\text {O}} \varvec{R}_i \varvec{I}^i_{\text {l}_i} \varvec{R}_i^\top J^{(\text {l}_i)}_{\text {O}} \dot{\varvec{q}}, \end{aligned}$$

where $m_{\text {l}_i}$ is the link mass, $\varvec{R}_i$ is the rotation matrix from Link *i* frame to the base frame, and $\varvec{I}^i_{\text {l}_i}$ is the inertia tensor relative to the centre of mass of Link *i* when expressed in the link frame (see Ch. 7 in [50]). The kinetic energy contribution of the motor of the *i*-th joint can be computed in an analogous way to that of the link.

Finally, by summing the contributions of the single links Eq. 16 and motors as in Eq. 11, the total kinetic energy of the manipulator is given by the quadratic form

17 $$\begin{aligned} K(\varvec{q}, \dot{\varvec{q}}) = \frac{1}{2} \sum _{i=1}^\alpha \sum _{j=1}^\alpha b_{ij}(\varvec{q})\dot{q_i} \dot{q}_j = \frac{1}{2} \dot{\varvec{q}}^\top \varvec{B}(\varvec{q}) \dot{\varvec{q}}, \end{aligned}$$

where $B(\varvec{q})$ is the inertia matrix (see Ch. 7 in [50]).

Let us now calculate the potential energy *P* of the manipulator as a function of the generalized coordinates. On the assumption of rigid links, the only conservative force is gravitational whose potential energy is given by

18 $$\begin{aligned} P(\varvec{q}) = - \sum _{i = 1}^\alpha (m_{\text {l}_i} \varvec{g}_0^\top \varvec{p}_{\text {l}_i} + m_{\text {m}_i} \varvec{g}_0^\top \varvec{p}_{\text {m}_i}), \end{aligned}$$

where $\varvec{g}_0$ is the gravity acceleration vector in the base frame, $m_{\text {m}_i}$ is the mass of the *i*-th motor, and $\varvec{p}_{\text {m}_i}$ is the position of the centre of mass of the motor in the base frame.

Let us now substitute the kinetic energy Eq. 17 and the potential energy Eq. 18 as functions of the generalized coordinates and velocities into the Lagrangian function Eq. 8 to get

19 $$\begin{aligned} L(\varvec{q}, \dot{\varvec{q}}) = K(\varvec{q}, \dot{\varvec{q}}) - P(\varvec{q}). \end{aligned}$$

Inserting Eq. 19 into the Lagrange’s equations of motion Eq. 10 and taking the required derivatives (note that *P* does not depend on $\dot{\varvec{q}}$) yields the standard form of robot dynamics Eq. 4 (see Ch. 7 in [50]). Therefore, Eq. 4 is a derivation of Eq. 10, and hence, it is also a derivation of Eq. 5.$\square$

### Design of Flexible Robotic Cell

A first decision affecting the design of the cell was which initial set of medical devices to work with while keeping the adaptability of the cell for processing other devices as an essential feature. We began by considering five models of inhalers for waste sorting and a model of glucose meter for disassembly. The five models of inhalers were chosen because they are quite different in terms of shape and color, and hence, suitable for appearance-based recognition algorithms as the one we were planning to use. At the same time, their significant differences would have demonstrated that the vision system of the cell can process various items and, with some tuning, be also able to detect other ones. The glucose meter, instead, was a good candidate for disassembly operations because the printed circuit board (PCB) contains electronic materials that are economically valuable, and hence, disassembling a large volume of them using robots justifies the operational costs involved in setting-up, running, and maintaining the disassembly cell.

**Fig. 1:**
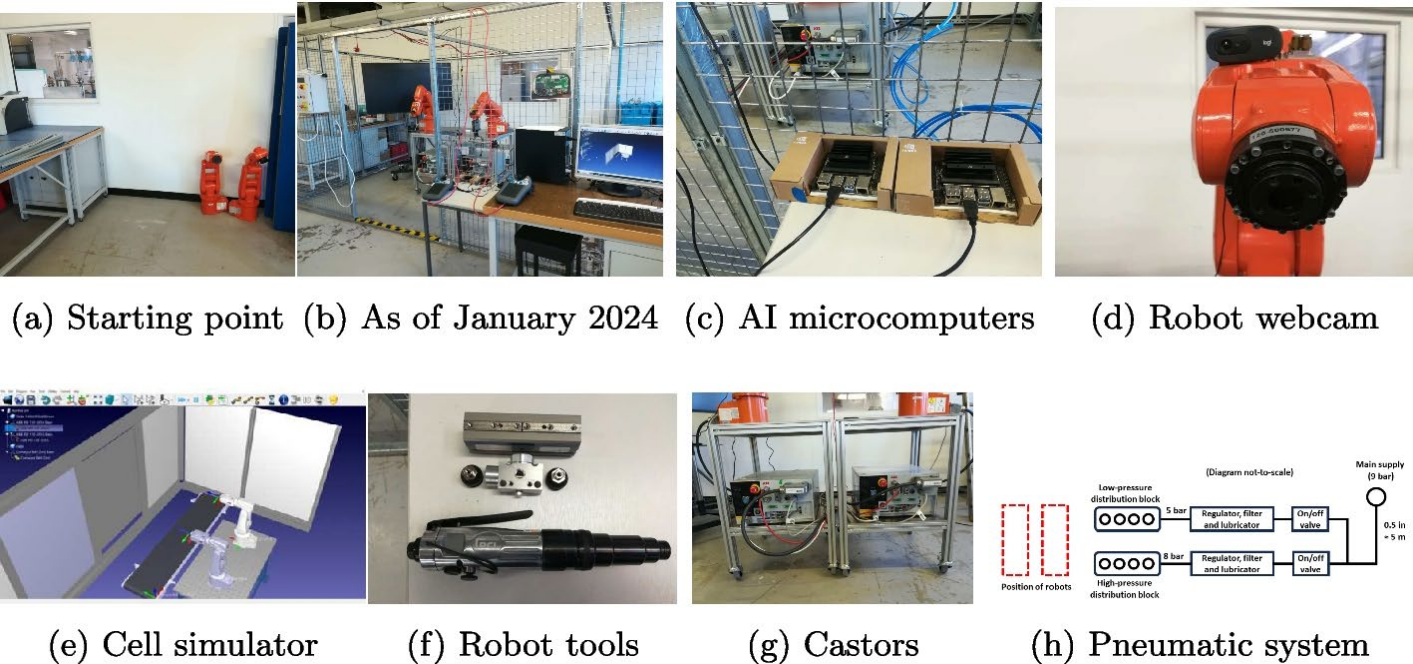
Main components of the flexible robotic cell

The main components of the flexible robotic cell we designed for reprocessing medical devices are shown in Fig. 1. An overview of our progress is visible by comparing Fig. 1a and 1b, which depict the initial and the current situation (as of January 2024), respectively. The cell occupies an area of 5 $\times$ 2.5 m and contains two ABB IRB 120 manipulators whose payload is 3 kg and whose reach is approximately 1.10 m (including the tool). The size of the robots has been chosen considering that the weight of the medical devices of our interest is less than 1.5 kg. These light robots are relatively easy to move to reconfigure the cell layout according to our research needs. The whole area is delimited by a safety cage since the ABB IRB 120 robot is not suitable to operate next to a human operator. A simulator of the robotic cell runs on the desktop beside the cage (bottom right of Figs. 1b and 1e) to check for collisions before operating the real robots and also to generate the robot programs. The simulator is developed on [52]. Each robot has a Logitech C270 webcam attached in proximity of its flange as in Fig. 1d. Each webcam is connected to an NVIDIA Jetson Nano microcomputer running deep neural networks for real-time image processing (Fig. 1c). Each robot is supported by a frame that contains the controller (model ABB IRC5) and it is installed on braked castors for cell reconfigurability (Fig. 1g). For example, waste sorting could be performed with the robots standing on the opposite sides of a conveyor belt, whereas disassembly could be performed with the robots facing each other. The robot tools are in Fig. 1f, they are pneumatic and a diagram of the air system is shown in Fig. 1h. Specifically, we have a vacuum gripper for quick and easy pick-and-place, a parallel gripper for more complex manipulations, a grinder and a screwdriver for semi-destructive and non-destructive disassembly operations, respectively.

The NVIDIA Jetson Nano microcomputer is a developer kit optimized for AI applications (Fig. 1c) [53]. We trained a ResNet-34 [54] on each microcomputer with batch size of 8 and the Adam optimizer [55] (we choose ResNet-34 after making the comparison with other neural models as reported in Table I in [56], whose this paper is a follow-up). One neural model was trained for 4 epochs on 266 images collected from the robot webcab (Fig. 1d) to classify syringes, glucose meters, and inhalers for waste sorting operations. The other model was trained for 10 epochs on 435 samples to track the position of the 3 screws that secure the printed circuit board (PCB) of a glucose meter to the plastic case. The application of this second vision system is screw removal either via a grinder (semi-destructive disassembly) or via a screwdriver (non-destructive disassembly).

### Circularity Indicators of Robotic Cell

**Fig. 2:**
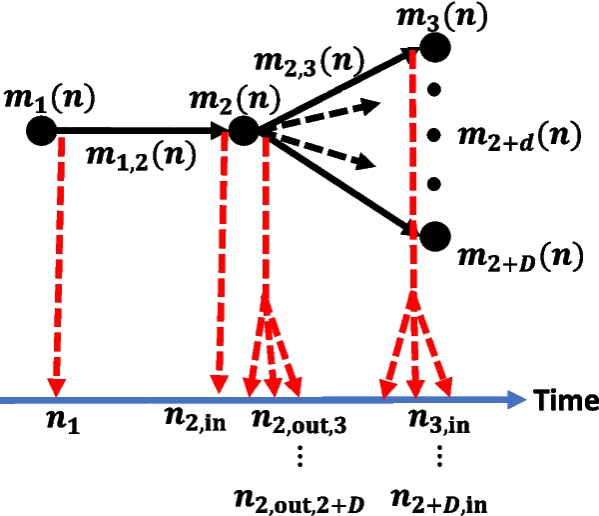
At the top, the mass-flow digraph of the robotic cell. The digraph is applicable to both disassembly and waste sorting scenarios. The red arrows indicate the corresponding time at which the mass leaves or enters a vertex-compartment.

To define the circularity indicators of the cell, first we model the cell as a mass-flow digraph $M(\mathcal {N})$ (Definition 4 in [10]), then we define the mass-flow matrix $\varvec{\Gamma }(\mathcal {N}; n)$ (Definition 1), and finally derive the indicators from the latter. The mass-flow digraph of the cell is shown in Fig. 2 and it is valid for both disassembly and waste sorting settings.

Specifically, in the case of disassembly, the digraph depicts the following scenario: $m_1(n)$ is the mass of a stock of products to be disassembled, $m_{1,2}(n)$ is the mass moved to the disassembly cell, $m_2(n)$ is the mass being disassembled, $m_{2,3}(n)$ is the mass of the materials moved to the first bin (case of $d = 1$), $m_{2,4}(n)$ is the mass of the materials moved to the second bin (case of $d = 2$), and $m_{2,2+d}(n)$ is the mass of the materials moved to the *d*-th bin. The total number of bins is *D*. Hence, $n_v = 2+D$, the number of arcs is $n_a = 1 + D$, and $n_c = 3 + 2D$. The mass leaves the first vertex (i.e., the stock of products) at time $n_1$, it enters the second vertex (i.e., the disassembly line) at time $n_{2,\text {in}}$, it leaves the second vertex at time $n_{2,\text {out},2+d}$ to enter the *d*-th bin, and it enters the *d*-th bin at time $n_{2+d,\text {in}}$. In contrast, in the case of waste sorting, the same digraph depicts the following scenario: $m_1(n)$ is a stock of waste to be sorted, $m_{1,2}(n)$ is the fraction of waste sent to the sorting cell, $m_2(n)$ is the mass inside the sorting cell, $m_{2,2+d}(n)$ is the mass of the materials moved to the *d*-th bin.

The TMN corresponding to the cell digraph (Fig. 2) is

20 $$\begin{aligned} \begin{gathered} \mathcal {N}^{\text {c}} = \left\{ c_{1,1}^1, c_{2,2}^2, \dots, c_{2+d,2+d}^{2+d}, \dots, c_{2+D,2+D}^{2+D}, c_{1,2}^{3+D}, \dots, \right. \\ \left. c_{2,2+d}^{3+D+d}, \dots, c_{2,2+D}^{3+2D} \right\}, \quad d = 1, \dots, D, \end{gathered} \end{aligned}$$

while the mass-flow matrix (Definition 1) becomes

21 $$\begin{aligned} \varvec{\Gamma }^{\text {c}}(\mathcal {N}; n) = \begin{bmatrix} m_1(n+1) & m_{1,2}(n+1) & \dots & 0 \\ 0 & m_2(n+1) & \dots & m_{2,2+D}(n+1) \\ \vdots & \vdots & \ddots \\ 0 & 0 & \dots & m_{2+D}(n+1) \end{bmatrix}, \end{aligned}$$

where $$\varvec{\Gamma }^{\text {c}}(\mathcal {N}; n) \in \mathbb {R}^{(2 + D) \, \times \, (2 + D)}$$ and $$n \in \overline{\mathbb {Z}}_+$$.

Let us now specify the entries of Eq. 21. The off-diagonal entries can be written as first order difference equations, that is,

22 $$\begin{aligned} m_{1,2}(n + 1) - m_{1,2}(n) = \overline{m}_{1,2} \left( \delta _{n_1}(n) - \delta _{n_{2,\text {in}}}(n)\right) \end{aligned}$$

and

23 $$\begin{aligned} \begin{gathered} m_{2,2+d}(n + 1) - m_{2,2+d}(n) = \overline{m}_{2,2+d} \left( \delta _{n_{2,\text {out},2+d}} \right. \\ \left. - \delta _{n_{2+d,\text {in}}} \right), \quad d = 1, \dots, D, \end{gathered} \end{aligned}$$

where

24 $$\begin{aligned} \delta _{n_1}(n) = {\left\{ \begin{array}{ll} 0, & \, n \ne n_1 \\ 1, & \, n = n_1, \end{array}\right. } \end{aligned}$$

which is the Kronecker delta. The definitions of the other Kronecker deltas are analogous to Eq. 24. The parameter $\overline{m}_{i,j}$ is the mass moving between vertex *i* and vertex *j* during the time window specified by the multiplying Kronecker deltas, e.g., for $\overline{m}_{1,2}$ in Eq. 22 the time window starts in $n_1$ and ends in $n_{2,\text {in}}$.

The entries along the main diagonal follow from the imposition of the principle of mass conservation in discrete time, that is,

25 $$\begin{aligned} m_1(n + 1) - m_1(n) = - \overline{m}_{1,2} \delta _{n_1}(n), \end{aligned}$$

26 $$\begin{aligned} \begin{gathered} m_2(n + 1) - m_2(n) = \overline{m}_{1,2} \delta _{n_{2,\text {in}}}(n) \\ - [\overline{m}_{2,3} \delta _{n_{2,\text {out},3}}(n) + \dots + \overline{m}_{2,2+d} \delta _{n_{2,\text {out},2+d}}(n) \\ + \dots + \overline{m}_{2,2+D} \delta _{n_{2,\text {out},2+D}}(n)]_{d = 2, \dots,D-1}\\ \end{gathered} \end{aligned}$$

and

27 $$\begin{aligned} m_{2+d}(n + 1) - m_{2+d}(n) = \overline{m}_{2,2+d} \delta _{n_{2+d,\text {in}}}(n), \quad d = 1, \dots, D. \end{aligned}$$

As the cell digraph is a closed system, from the principle of conservation of mass it follows that

28 $$\begin{aligned} \sum _{i=1,j = 1}^{2+D} \gamma ^{\text {c}}_{i,j}(n) = \text {const.} \quad \forall n, \end{aligned}$$

where $\gamma ^{\text {c}}_{i,j}$ is the (*i*, *j*) entry of $\varvec{\Gamma }^{\text {c}}$.

The mass-flow matrix Eq. 21 contains useful information related to the material circularity: the stock masses are along the main diagonal, the mass flows are off-diagonal, the times $n_1$, $n_{2,\text {in}}$, etc. at which a certain mass enters or leaves a stock is expressed by each entry, and it also contains the information about the layout of the cell, e.g., the number of rows of $\varvec{\Gamma }^{\text {c}}(\mathcal {N}; n)$ depends on the number of bins *D*, while the entry $\gamma _{i,j}(n)$ indicates the material flow from the vertex *i* to the vertex *j*; if $\gamma _{i,j}(n) = 0$, the two vertices are not exchanging mass directly (for example, the stock $m_1$ and the first bin $m_3$).

We now define two circularity indicators for the robotic cell using the mass-flow matrix Eq. 21. The first indicator is the *separation rate* $r_\text {s}$ defined as

29 $$\begin{aligned} r_\text {s} \triangleq \text {Number of rows of }\, \varvec{\Gamma }^{\text {c}}(\mathcal {N}; n), \end{aligned}$$

which quantifies to what extent the cell separates the input flow $m_{1,2}(n)$ into different flows $m_{2,2+d}(n)$ to facilitate the recovery of the materials or the parts. An increase in the number of bins *D* yields an increase of $r_\text {s}$ since $r_\text {s} = 2 + D$. The second circularity indicator is the *separation time* $t_\text {s}$ defined as

30 $$\begin{aligned} t_\text {s} \triangleq \max _{\begin{array}{c} d \\ 1 \le d \le D \end{array}} \left( n_{2,\text {out},2+d} - n_{2,\text {in}}\right) T, \end{aligned}$$

where *T* is the sample time at which the dynamics of the system is recorded. In words, $t_\text {s}$ is the time that passes from when $\overline{m}_{1,2}$ enters the robotic cell (i.e., vertex 2) to when the *last* mass leaves the robotic cell to enter the designated *d*-th bin (i.e., vertex $2+d$). With respect to the circularity of material flows, the indicators $t_\text {s}$ and $r_\text {s}$ reward a robotic cell that is both fast (i.e., $t_\text {s}$ is low, e.g., $t_\text {s} < 2$ minutes) and able to accurately separate many components (i.e., $r_\text {s}$ is high, e.g., $r_\text{s}> 5$), which facilitates the recovery of parts and materials for future reuse or recycling. Note that optimizing the two indicators simultaneously is challenging because an increase of $r_\text {s}$ will likely result in an increase of $t_\text {s}$; to keep $t_\text {s}$ low, the robot speed should be increased, but this may result in failing a disassembly step, which yields a reduction in the number of parts or materials separated successfully (i.e., $r_\text {s}$ decreases).

#### Remark 1

The indicators $r_\text {s}$ and $t_\text {s}$ could have been defined without introducing $\varvec{\Gamma }^{\text {c}}(\mathcal {N}; n)$. However, the advantage of defining the circularity indicators from $\varvec{\Gamma }^{\text {c}}(\mathcal {N}; n)$, and hence, from a digraph, is in the generality of the approach. Indeed, the same graph-based procedure can be used to develop circularity indicators in continuous-time (e.g., see [40]), in discrete-time (e.g., in this paper), with smaller networks (e.g., the robotic cell covered here) and with larger networks such as the supply chains (e.g., see [10]).

#### Remark 2

From Definition 2, it follows that the cell is *not thermodynamically circular* since the cell digraph (Fig. 2) has not any $\phi$, i.e., it has no closed loops. This matches with the reality since a robotic cell cannot close the material flow on its own; it can do so if it is part of a recovery chain that involves also the transportation and reuse of the materials or products.

#### Remark 3

The systemic framework leveraged in this paper enhances MFA as it is not merely based on mass balances, but it also considers the energy balances (i.e., the first principle of thermodynamics). Specifically, in this paper, the first principle of thermodynamics was covered in Section 2.1 to derive the mechanics of robots, while the mass balances were used for deriving the circularity indicators in Section 2.3.

#### Remark 4

The circularity indicator Eq. 29 is defined within the TMN methodology because it is computed from the mass-flow matrix $\varvec{\Gamma }(\mathcal {N}; n)$ in Eq. 2, which is associated with a TMN by definition (Definition 1). The indicator Eq. 30 is also defined within the TMN methodology because it results from the mass-flow digraph in Fig. 2 and the mass-flow digraph represents a TMN by definition (Definition 4 in [10]).

## Results and Discussion

This section covers the current capabilities of the flexible robotic cell. Then, it illustrates a numerical example about the robotic cell circularity.

### Current Functionality of Robotic Cell

**Fig. 3:**
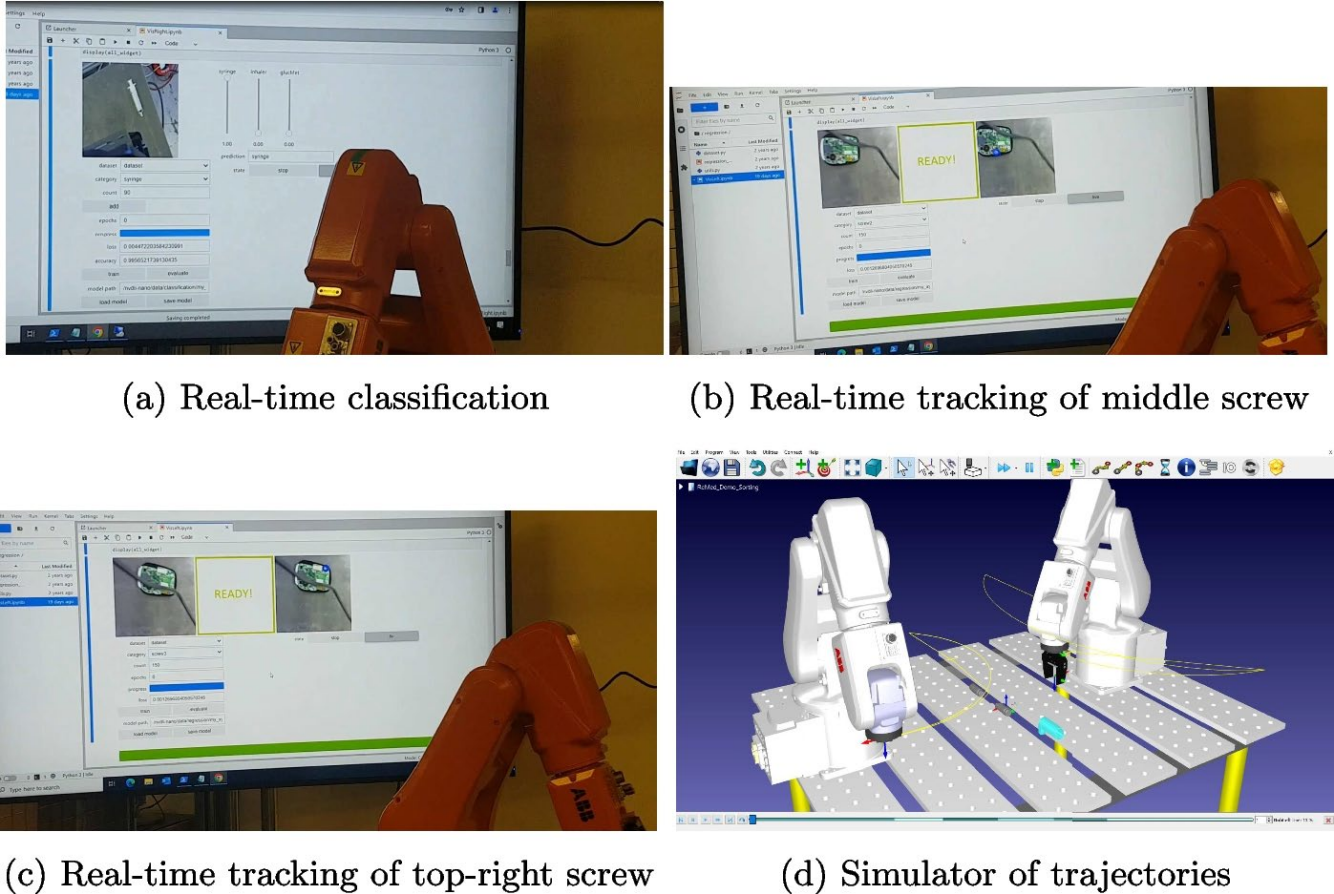
Current functionalities of the flexible robotic cell. Inhaler CAD model taken from [57]

The current functionalities of the cell are depicted in Fig. 3.

Specifically, Fig. 3a shows the camera view (top left) and the correct classification of a syringe indicated by the cursor (next to camera view). The neural model has 99% accuracy on collected images (bottom left). This picture is taken while the robot is programmed to autonomously move above the syringe; simultaneously, the webcam sends the frames to a Jetson Nano, which then performs the recognition of the object in real-time. Note that classification and object detection are two well-distinct tasks in computer vision; specifically, the latter is an evolution of the former since it also provides information about the position of the object. At this initial stage, we began with real-time classification with the plan of increasing in complexity and functionality at a later time. Indeed, in subsequent work, we implemented real-time object detection for processing inhalers as detailed in [58]. Recognizing objects is an essential step for both waste sorting and disassembly operations.

In contrast, Figs. 3b and 3c show tasks that are useful mainly for disassembly. Specifically, Fig. 3b shows the real-time tracking of a screw indicated by the blue circle even with the disturbance of a cable, while Fig. 3c considers another screw. These screws secure the glucose meter PCB to the plastic case. The manipulators are programmed to move autonomously, the programs are exported from RoboDK and loaded into the IRC5 robot controllers. Unscrewing is an important task in disassembly since it is common to find products with screws. It requires an accurate positioning of the object and the robot tool. When such accuracy is not achievable, an alternative approach is destructive disassembly, which permanently damages the device, e.g., using a grinder to cut the area around the screw. While screwing and unscrewing a device with a well-known geometry is a standard step in industry, here we are using a vision system that recognizes the screw and tracks its position in real-time. This vision-based approach makes the robotic operation more flexible than without it because it can provide the position of screws located at unknown points. In contrast, standard automation programs require the “blind” robot to move to the known position of the screw. If there is a significant error in the tool-screw alignment, the unscrewing step fails. Using vision as we show here not only can solve potential misalignments, but also it could unscrew at points that are not explicitly inserted into the robot program because the position of the screw is provided by the vision algorithm that processes the camera frames in real-time. Note that our vision-based unscrewing operation is not functional yet; these experiments, i.e., Figs. 3b and 3c, demonstrate the visual tracking of two screws on this particular PCB.

Figure 3d depicts the RoboDK simulator. The robot trajectories are shown in yellow and they enable the robot programmer to visualize the robot behavior before running the program in the real machine. The importance of having a simulator of the cell is to check if a given program causes collisions between the components of the cell, e.g., between the two robots or between a robot end effector and the bench. Another benefit specific of RoboDK is that it does not require knowledge of the programming language of the robot in order to simulate it. This can be time saving since it is common that robot manufacturers develop their own programming language. With RoboDK, once the programmer is satisfied with the simulation behavior, the robot program can be immediately exported in the robot relevant language so that it can be uploaded to the robot controller for execution. The current limitation of our simulator is that it does not communicate with the Jetson Nano, and hence, it programs the robot without the use of visual inputs, i.e., using the standard non-adaptive approach of coding explicitly the desired points to be reached by the robot. A demo video of these experiments is available^1^.

Medical devices show a significant variability in their designs, which result in different shapes, colors, parts, and disassembly sequences. Currently, the robotic cell is at its initial development stage, and hence, it cannot process all types of devices. The diversification of the types of processed devices can be increased by training the vision algorithm on images of new devices, changing or designing the robot end effectors to handle the new devices, changing the robot program in order to perform the disassembly sequence suitable for the new devices, and changing the gripping system that holds the device during disassembly. Hence, the flexibility of the cell is mainly affected by four components: the vision system, the end effector, the robot program, and the gripping system. The same considerations hold for waste sorting with the exceptions that: first, in waste sorting there is no gripping system, and second, a vacuum gripper is often the most effective end effector because of the simplicity of the grasping task [19]. The castors enable us to create configurations that are more suitable for waste sorting or disassembly, e.g., robots facing each other with a disassembly bench in the middle, robots on the same side of a conveyor belt.

To perform resources mapping and quantification, we recently provided in [58] a first small prototype implemented in this flexible robotic cell. The prototype detects 4 materials from 5 different models of inhalers and, through a synchronization mechanism, it combines the detection outputs of the 2 microcomputers in Fig. 1c, each one running a deep-learning object detector at 12-22 frames per second. The dataset used for training both the detectors contained a total of 1000 fully-annotated images with 6 classes (refer to [58] for details). To highlight the synchronization mechanism needed to compute the total mass of the materials detected at different point of the robotic cell, we also introduced the notion of *synchromaterial* in [58].

### Numerical Example on Cell Circularity

To illustrate the practical use of $\varvec{\Gamma }^{\text {c}}$, $r_\text {s}$, and $t_\text {s}$, let us assume the following scenario: a GlucoRx HCT glucose meter has to be disassembled by a robotic cell; the masses of its parts are given in Table 1.

**Table 1:** Masses of the parts of the considered glucose meter and the bin indices

Part	Mass	*d*
Front case	14.4 g	1
Back case	17.2 g	1
PCB	17.8 g	2
Screw	0.2 g	3
Spring	0.1 g	4
Button and clip	1.6 g	4
Test strip port	1.0 g	4
Screen	8.4 g	4
USB port cap	0.3 g	4
Whole ($m_{\text {gm}}$)	61.8 g	-

The layout of the cell has 4 bins: the first bin is for the casing, the second bin is for the printed circuit board (PCB), the third bin is for the 5 screws, and the fourth bin is for any other parts. Hence, in this case, $D = 4$. Let us assume also that the situation is monitored every second, i.e., $T = 1$ s. The times at which the masses are moved are: the glucose meter enters the disassembly line at $n_{2,\text {in}} = 30$ after being extracted from a stock of 2 glucose meters at $n_1 = 5$; the casing, the PCB, the screws, and the remaining parts leave the disassembly line at $n_{2,\text {out},3} = 240$, $n_{2,\text {out},4} = 300$, $n_{2,\text {out},5} = 320$, and $n_{2,\text {out},6} = 360$, respectively. The casing, the PCB, the screws, and the remaining parts enter the designated bin at $n_{d,\text {in}} = n_{2,\text {out},d} + 5$, that is, the transfer from the disassembly line to the *d*-th bin takes 5 sample times for $d = 1, \dots, 4$. This transfer time is the time that it takes a robotic arm to pick a disassembled part (e.g., the PCB) and place it into the designated bin. Thus, the time for separating a part from the glucose meter is different from the time needed for moving the same part to the bin; the former is, in general, significantly longer than the latter.

With this scenario, the mass-flow matrix of the robotic cell Eq. 21 becomes

31 $$\begin{aligned} \varvec{\Gamma }^{\text {c}}(\mathcal {N}; n) = \begin{bmatrix} \theta _0 & \theta _1 & 0 & 0 & 0 & 0 \\ 0 & \theta _2 & \theta _3 & \theta _4 & \theta _5 & \theta _6 \\ 0 & 0 & \theta _7 & 0 & 0 & 0 \\ 0 & 0 & 0 & \theta _8 & 0 & 0 \\ 0 & 0 & 0 & 0 & \theta _9 & 0 \\ 0 & 0 & 0 & 0 & 0 & \theta _{10} \end{bmatrix}, \end{aligned}$$

where

32 $$\begin{aligned} \theta _0 = m_1(n) - \overline{m}_{1,2} \delta _{n_1}(n), \end{aligned}$$

33 $$\begin{aligned} \theta _1 = m_{1,2}(n) + \overline{m}_{1,2} \left( \delta _{n_1}(n) - \delta _{n_{2,\text {in}}}(n)\right), \end{aligned}$$

34 $$\begin{aligned} \begin{gathered} \theta _2 = m_2(n) + \overline{m}_{1,2} \delta _{n_{2,\text {in}}}(n) - \overline{m}_{2,3} \delta _{n_{2,\text {out},3}}(n) \\ - \overline{m}_{2,4} \delta _{n_{2,\text {out},4}}(n) - \overline{m}_{2,5} \delta _{n_{2,\text {out},5}}(n) - \overline{m}_{2,6} \delta _{n_{2,\text {out},6}}(n), \end{gathered} \end{aligned}$$

35 $$\begin{aligned} \theta _3 = m_{2,3}(n) + \overline{m}_{2,3} \left( \delta _{n_{2,\text {out},3}} \right. \left. - \delta _{n_{3,\text {in}}} \right), \end{aligned}$$

36 $$\begin{aligned} \theta _4 = m_{2,4}(n) + \overline{m}_{2,4} \left( \delta _{n_{2,\text {out},4}} \right. \left. - \delta _{n_{4,\text {in}}} \right), \end{aligned}$$

37 $$\begin{aligned} \theta _5 = m_{2,5}(n) + \overline{m}_{2,5} \left( \delta _{n_{2,\text {out},5}} \right. \left. - \delta _{n_{5,\text {in}}} \right), \end{aligned}$$

38 $$\begin{aligned} \theta _6 = m_{2,6}(n) + \overline{m}_{2,6} \left( \delta _{n_{2,\text {out},6}} \right. \left. - \delta _{n_{6,\text {in}}} \right), \end{aligned}$$

39 $$\begin{aligned} \theta _7 = m_{3}(n) + \overline{m}_{2,3} \delta _{n_{2,\text {out},3}}(n), \end{aligned}$$

40 $$\begin{aligned} \theta _8 = m_{4}(n) + \overline{m}_{2,4} \delta _{n_{2,\text {out},4}}(n), \end{aligned}$$

41 $$\begin{aligned} \theta _9 = m_{5}(n) + \overline{m}_{2,5} \delta _{n_{2,\text {out},5}}(n), \end{aligned}$$

and

42 $$\begin{aligned} \theta _{10} = m_{6}(n) + \overline{m}_{2,6} \delta _{n_{2,\text {out},6}}(n). \end{aligned}$$

Moreover, the circularity indicators of the cell become $t_\text {s} = (n_{2,\text {out},6} - n_{2,\text {in}})T = 330$ s and $r_\text {s} = 6$ (from Eqs. 30 and 29, respectively).

**Fig. 4:**
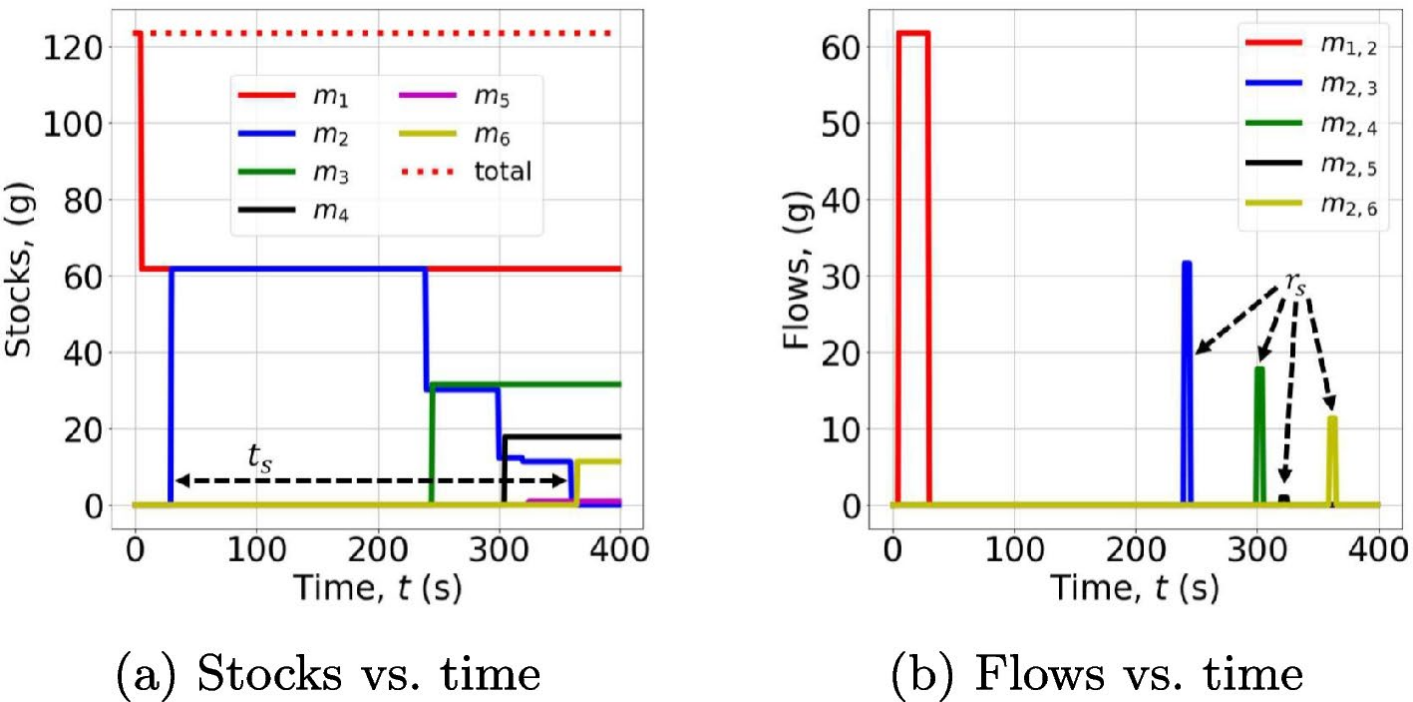
Dynamics of the stocks and flows of the robotic cell, which correspond to the entries $\theta _0, \theta _1, \dots, \theta _{10}$ of the mass-flow matrix Eq. 31

Now, let us analyze the dynamics of stocks and flows within the system, which are shown in Fig. 4. Then, we will analyze how they affect the circularity indicators $t_\text {s}$ and $r_\text {s}$. As visible in Fig. 4a, $m_1$ is the only stock for $t < n_1 = 5$ s, whose value is $m_1 = 2m_{\text {gm}} = 123.6$ g. Then, as visible in Fig. 4b, $m_{1,2} = m_{\text {gm}} = 61.8$ g for $n_1 \le t < n_{2,\text {in}}$. For $n_{2,\text {in}} \le t < n_{2,\text {out},3}$, it holds that $m_2 = m_{\text {gm}}$. In particular, the dynamics of $m_2$ has 5 variations: the first variation is in $t = n_{2,\text {in}}$ and it is an increase of mass because $m_{\text {gm}}$ enters, whereas the other 4 variations are reductions because the mass exits to enter the 4 designated bins. These 4 mass transfers can be seen easily in Fig. 4b as they correspond to the 4 peaks in $m_{2,3}$, $m_{2,4}$, $m_{2,5}$, and $m_{2,6}$. These 4 peaks are rectangles with duration of 5 s, which is the transfer time of the glucose meter parts from the disassembly line ($m_2$) to any bin. The tallest rectangle in Fig. 4b is $m_{1,2}$, which is the transfer of the glucose meter (61.8 g) from the stock $m_1$ to the disassembly line $m_2$. Note also from Fig. 4a that $m_3$, $m_4$, $m_5$, and $m_6$ have an increase with the same magnitude and immediately after the corresponding decrease of $m_2$ since the disassembled parts exit $m_2$ to enter $m_3$, $m_4$, $m_5$, and $m_6$. The third bin (i.e., $m_5$) receives the 5 screws, hence it contains the smallest mass, that is, $5 \times 0.2 = 1$ g. As the whole robotic cell is a closed system, the total mass within it is constant as showed in Fig. 4a and it is equal to $2 \times m_{\text {gm}} = 123.6$ g.

The performance of the robotic cell in terms of circularity is measured by the indicators $t_\text {s}$ Eqs. 30 and $r_\text {s}$ 29. The former is indicated in Fig. 4a and it is affected by the speed of the disassembly, which can be increased, for example, by speeding-up the disassembly sequence or by reducing the image processing latency. The latter is indicated in Fig. 4b and, in contrast with $t_\text {s}$, an higher value leads to a better cell from the circularity perspective. Indeed, a cell with higher $r_\text {s}$ can separate more materials to facilitate their reuse or recycling. Intuitively, $r_\text {s}$ can be increased by increasing the number of bins *D* since $r_\text {s} = 2+D$. However, this may lead to a more complex disassembly sequence, and hence, it may increase $t_\text {s}$ (e.g., with $D = 1$ no disassembly is performed since the product goes straight into the only bin; if $D = 1$, $r_\text {s} = 3$, which is the worst value, whereas $t_\text {s} = 0$ s, which is ideal). Therefore, improving $r_\text {s}$ and $t_\text {s}$ jointly can be particularly challenging. In this case, $t_\text {s} = 330$ s and $r_\text {s} = 6$.

The two circularity indicators can help decision-making in planning recovery chains in healthcare. Specifically, $t_\text {s}$ gives information about the time that the disassembly or sorting operation takes for a single product. Multiplying $t_\text {s}$ by the total number of products to be processed yields the time taken for processing all the products. The manager of the recovery chain can then assess whether the time $t_\text {s}$ is acceptable or, if too high, consider to change the setup of the robotic cell to increase its speed (e.g., increase the speed of robots or purchase another robot). In parallel, $r_\text {s}$ informs the manager whether the cell divides the input stream of material into a sufficient number of sub-streams; for example, if the goal is to recover a PCB and the metal from the screws of a glucose meter, it must be $r_\text {s} \ge 5$ ($D = 3$) so that one bin is for the PCB, one bin is for the screws, and a bin is for anything else. If $r_\text {s} < 5$, the cell does not sufficiently separate the input stream of material and interventions must be made in the cell to increase $r_\text {s}$, e.g., the program for the disassembly sequence must perform a more granular separation of the parts. Note that, while the time required by the robotic operation, namely, $t_\text {s}$, is a common performance indicator in robotics, $r_\text {s}$ is meaningful in the specific context of circular economy since the aim is to recover the materials by properly separating them. Separating product parts and materials is unnecessary if they are sent to a landfill or an incinerator afterwards (linear economy).

## Conclusions

This paper presented the development of a flexible robotic cell for reprocessing small medical devices without contamination risks and also proposed two indicators to measure the robotic cell circularity leveraging a thermodynamics-based systemic modeling framework for circular flow designs of materials.

As of January 2024, the phase of building the robotic cell is almost complete, whereas the phase of programming remains in progress. Indeed, as soon as the communication between the microcomputers and the manipulators is established, the visual inference should provide a real-time and accurate feedback to the robot controllers to achieve a human-like adaptive behavior. A key challenge will be to find a compromise between the visual feedback accuracy, the computation latency, and the communication latency to effectively perform waste sorting and disassembly operations of items located in random positions. Further development of the robotic cell and testing of its performance will enable us to measure how many devices can be processed per unit of time, e.g., per day, and hence, to properly assess its suitability as a reprocessing stage of a recovery chain in real-world healthcare settings. Importantly, the number of devices to be processed each day by the cell in real recovery chains depends on the extent to which circular practices are adopted in the healthcare sector. The extent to which circular practices are adopted affects the scalability of robotic and, more generally, autonomous solutions for waste and material reprocessing. Indeed, the costs of purchasing, operating, and maintaining robots is justifiable only if the volume of products to be reprocessed is sufficiently large. If the future is really going to be more circular, these volumes are going to be significant, and hence, robotics and automation will certainly have a key role to play as it happened in linear supply chains over the last century.

The integration of robot dynamics into a systemic modeling framework enables us to embed the modeling and control of industrial manipulators into the design of recovery chains, and thus, to analyze and regulate the robot performance with respect to the material flow circularity indicators. This integration is important to create accurate physics-based models of supply-recovery chains, which are needed to design circular flows of materials and products. A robot used for waste sorting or disassembly is part of the recovery chain and the accurate modeling developed by the robotics community over the years “enters” our thermodynamic framework as a thermodynamic compartment. Other compartments are, for example, transport vehicles (whose modelling can be derived from the Lagrange’s mechanics, which in turn, derives from the first law of thermodynamics as demonstrated with Proposition 1 in [10]). Data-analysis approaches to circular economy modelling such as MFA do not include robot modeling because they are not based on dynamical systems theory and the laws of thermodynamics. Our approach, instead, does so by leveraging compartmental dynamical thermodynamics [10, 11]. Note also that both dynamical systems theory and the first law of thermodynamics are necessary to coherently integrate robot modeling into supply-chain models. This is visible from Proposition 1.

The numerical study on circularity indicators highlighted that the separation time, that is, the robot speed, is a key factor as in the manufacturing processes of linear economies. However, differently from manufacturing, the rate of material separation, i.e., $r_\text {s}$, is a key factor for reprocessing operations since it facilitates the reuse of parts, the repair of products, and the material recycling.

This paper also has coherently integrated deep-learning vision, robotics theory, thermodynamics, and graph theory to make a step forward in the definition of the theoretical foundations of circular economy as a scientific discipline. Indeed, while the behavioral principles for improving circularity are clear, i.e., reduce, reuse, repair, recycle, etc., the actual design of circular material flows is currently lacking the mathematical rigor existing in other more mature fields such as electrical network design [59], robot design [50], vehicle design [60], and artificial neural network design [61]. The mathematical rigor results in models that are both more accurate and reproducible.

This research has two main limitations. First, the robotic cell is not yet performing a completely autonomous disassembly or sorting operation leveraging the visual inputs coming from the Jetson Nano microcomputer; this is because, as mentioned above, the communication between the robot controllers and the Jetson Nano has yet to be established and tested. Second, the thermodynamic framework requires the correct application of the laws of thermodynamics; although applied thermodynamics is a well-established subject in physics and engineering, its use to study new systems, such as circular flows, can become challenging, especially to model real large supply-recovery chains. In general, the modeling accuracy comes at the cost of some complexity. This is visible, for example, by looking at how the design of artificial neural networks has evolved in the last thirty-five years, with the result that now there are highly skilled researchers and engineers employed in industry specifically to develop neural learning systems able to perform tasks that were not possible back in the nineties.

Future work regarding the cell will be to enable the communication between the AI microcomputers and the manipulator controllers to synchronize the visual inference with the robot dynamics. After that, for waste sorting, it will be added a conveyor belt to the cell since, at the time of writing, the conveyor belt is included only in the simulator (Fig. 1e). For disassembly, the sequence of actions for removing the parts of a glucose meter will be identified and a gripping system for holding it will be added to the cell. For performing autonomous resources mapping and quantification, the performance of the first prototype recently proposed in [58] could be improved. Finally, we must investigate how the robotic cell and the thermodynamic framework are affected by healthcare regulations in order to comply with them.

## Data Availability

Software and demo video are available at: https://github.com/fedezocco/ThermoVisMedRob.

## Footnotes

1 https://github.com/fedezocco/ThermoVisMedRob
